# The Efficiency and Safety of Mirabegron Monotherapy for the Treatment of Urge Incontinence in Women Aged >80 Years

**DOI:** 10.7759/cureus.33685

**Published:** 2023-01-12

**Authors:** Cihat Özcan, Adem Sancı, Murat Beyatlı, Selahattin Bedir, Yaşar Özgök

**Affiliations:** 1 Department of Urology, Health Sciences University, Gülhane Training and Research Hospital, Ankara, TUR; 2 Department of Urology, Etlik City Hospital, Ankara, TUR; 3 Department of Urology, Sakarya Yenikent State Hospital, Sakarya, TUR

**Keywords:** drug side effect, elderly population, women, urge incontinence, mirabegron

## Abstract

Objective

We aimed to evaluate the efficacy and safety of mirabegron monotherapy in very older (>80 years) women with overactive bladder (OAB) who were discontinued anticholinergic drugs by the other departments.

Material and methods

The present retrospective study evaluated very older (>80 years) women with OAB who were discontinued anticholinergic drugs by the other departments between May 2018 and January 2021. Efficacy assessments were performed using Overactive Bladder-Validated Eight-Question (OAB-V8) scores before and after mirabegron monotherapy (12 weeks). Safety was evaluated based on adverse events (hypertension, nasopharyngitis, and urinary tract infection), electrocardiography, hypertension measure, uroflowmetry (UFM), and post-voiding. Patient data including demographic characteristics, diagnoses, values before and after mirabegron monotherapy, and adverse events were evaluated.

Results

A total of 42 very older (>80 years) women with OAB who used mirabegron monotherapy (50 mg per day) were included in this study. Frequency, nocturia, urgency, and total OAB-V8 scores were significantly lower after mirabegron monotherapy than before mirabegron monotherapy (p < 0.05, p < 0.05, p < 0.05, and p < 0.05, respectively). There was no significant difference between systolic-diastolic blood pressure and heart rate before and after mirabegron monotherapy treatment.

Conclusion

Mirabegron monotherapy is an effective and safe therapy in very older (>80 years) women with OAB.

## Introduction

The International Continence Society (ICS) defines urinary incontinence (UI) as involuntary urinary leakage [1]. Urinary incontinence (UI) may associate with dysfunctions of the lower urinary system or other diseases. Pregnancy, multiparity, obstetric causes, menopause, hysterectomy, obesity, smoking, family history, diet, and genetic causes are the risk factors for UI [2]. The types of UI include the following: stress urinary incontinence (SUI), urge urinary incontinence (UUI), mixed urinary incontinence (MUI), and overactive bladder (OAB) [1]. The incidence of UUI and OAB, which seriously affects social life in women, increases with advanced age [3].

Anticholinergic (antimuscarinic) drugs and beta-3 agonists are currently the main pharmacological management for OAB. Beta-3 adrenoceptors are the dominant beta receptors that are expressed on detrusor muscle cells [4,5]. Beta-3 receptor stimulation is associated with inducing detrusor relaxation. Mirabegron is the first beta-3 agonist. Vibegron is another beta-3 agonist used in some regions [4,6]. The 2022 European Association of Urology (EAU) panel suggests that if an anticholinergic treatment proves ineffective, consider dose escalation, offering an alternative anticholinergic formulation, or the use of mirabegron (alone or in combination with an anticholinergic) [7]. The adverse effects (dry mouth, constipation, and cognitive dysfunction resulting in discontinuation of treatment) of antimuscarinic treatment for OAB generally do not occur in the use of mirabegron. Some authors suggested that mirabegron should be started primarily in elderly patients with high anticholinergic load due to polypharmacy [8]. In older patients, frailty is a global health problem that is associated with OAB. Although analysis of data from some randomized controlled trials (RCTs) showed that mirabegron was effective and safe in elderly patients, it is still no consensus, especially in very older women who are required to discontinue the anticholinergic drugs [9,10]. In older women, there are little data about whether beta-3 adrenoceptor agonists trigger adverse events when used to treat OAB [10].

In this study, we aimed to evaluate the efficacy and safety of mirabegron monotherapy in very older (>80 years) women with OAB who were discontinued anticholinergic drugs by the other departments.

## Materials and methods

The present retrospective study evaluated very older (>80 years) women with OAB who were discontinued anticholinergic drugs by the other departments between May 2018 and January 2021. For the study, ethical approval was obtained from our local ethical committee (number: 2022/113).

The inclusion criteria were as follows: patients who are >80 years old, presence of complaints for at least three months, presence of uncomplicated OAB symptoms (not requiring invasive urodynamic tests including multichannel cystometry and pressure flow studies, ambulatory monitoring, and video urodynamics), presence of a history of using at least one anticholinergic alone or as part of combination therapy and the discontinue of the anticholinergic drugs by the other departments (internal medicine, neurology, and geriatric) due to drug interaction, increase in side effect profile, inability to comply with medication, presence of the history using mirabegron (50 mg per day) alone for at least 12 weeks after the discontinued anticholinergic therapy, presence of an evaluation using the Overactive Bladder-Validated Eight-Question (OAB-V8) form before and after medication.

The cutoff age was 80 years and over because the current literature used this value [10,11]. Efficacy assessments were performed using OAB-V8 scores before and after mirabegron monotherapy (12 weeks) [12]. Safety was evaluated based on adverse events (hypertension, nasopharyngitis, and urinary tract infection), electrocardiography, and hypertension measure. None of the patients in the study did have a Mini-Mental State Examination (MMSE) score. It may be explained by the retrospective nature of the study. Information about the patients was collected from the medical records system of the hospital. Patient data including demographic characteristics, diagnoses, values before and after mirabegron monotherapy, and adverse events were evaluated.

Statistical analysis

All data in both groups were compared using Statistical Package for Social Sciences (SPSS) version 25.0 (IBM SPSS Statistics, Armonk, NY, USA) for Windows. Continuous variables were represented as mean ± standard deviation and median ± range (minimum-maximum), and categorical variables were represented as numbers and percentages. A chi-squared test (Fisher’s exact test) was used to compare categorical variables (before and after mirabegron monotherapy). The Mann-Whitney U test was used for non-categorical variables. p < 0.05 was considered to be the threshold for statistical significance.

## Results

A total of 42 very older (>80 years) women with OAB who were discontinued anticholinergic drugs by the other department and who used mirabegron monotherapy (50 mg per day) were included in this study.

The median age was 86 years (range: 80-93 years). The women in the study had a median of 4 (range: 2-6) comorbid conditions. The median number of concomitant medications of the patients in this study was 6 (range: 3-9). The median symptom score (according to the OAB-V8 form) of the patients was 19 (range: 10-28). The median voiding volume according to uroflowmetry (UFM) was 127 mL (range: 100-148 mL). The median maximum flow rate was 12 mL/second (range: 9-16 mL/second). The median post-void residual (PVR) was 16 mL (range: 6-58 mL). The median heart rate was 64 beats/minute (range: 57-73 beats/minute). The median systolic and diastolic blood pressure were 129 and 82 mmHg, respectively. The patients’ demographic characteristics are summarized in Table 1.

**Table 1:** Baseline patient characteristics OAB: overactive bladder, OAB-V8: Overactive Bladder-Validated Eight-Question, PVR: post-void residual

Characteristic	Very older (>80 years) women with OAB (n = 42)
Age, median (range)	86 (80-93)
Comorbid conditions, median (range)	4 (2-6)
Concomitant medications, median (range)	6 (3-9)
Symptom score (OAB-V8 form), median (range)	19 (10-28)
Voiding volume (mL), median (range)	127 (100-148)
PVR volume (mL), median (range)	16 (6-58)
Maximum flow rate (mL/second), median (range)	12 (9-16)
Heart rate (beats/minute), median (range)	64 (57-73)
Systolic and diastolic blood pressure (mmHg)	129 (95-145) and 82 (65-104)

Frequency, nocturia, urgency, and total OAB-V8 scores were significantly lower after mirabegron monotherapy than before mirabegron monotherapy (p < 0.05, p < 0.05, p < 0.05, and p < 0.05, respectively). There was a significant statistical difference in the symptom scores before and after mirabegron monotherapy (p = 0.023). The effectiveness outcomes and OAB-V8 form scores are summarized in Table 2.

**Table 2:** Changes from baseline to the end of mirabegron monotherapy

	Before treatment	After treatment	p value
Symptom scores, median (range)	19 (10-28)	10 (7-14)	<0.05
Urgency	6 (4-9)	3 (2-6)	<0.05
Frequency	12 (8-17)	7 (5-11)	<0.05
Nocturia	2 (1-5)	1 (1-2)	<0.05

There was no significant difference between systolic and diastolic blood pressure and heart rate before and after mirabegron monotherapy treatment. Renal failure, liver transaminase elevations, QT prolongation, irregular heart rhythms, urinary retention, and drug interaction (digoxin, dabigatran, and antibiotics) did not occur in any patients. The adverse events and safety criteria were summarized in Table 3 and Table 4.

**Table 3:** Adverse events of mirabegron monotherapy QT prolongation did not occur in any patient.

Data	Before treatment	After treatment	p value
Heart rate (beats/minute), median (range)	64 (57-73)	66 (53-82)	0.48
Systolic blood pressure (mmHg), median (range)	129 (95-145)	127 (96-149)	0.32
Diastolic blood pressure (mmHg), median (range)	82 (65-104)	85 (68-105)	0.11

**Table 4:** Adverse events of mirabegron monotherapy OAB: overactive bladder

Adverse events	Very older (>80 years) women with OAB (n = 42)
Dry mouth	2 (4%)
Headache	1 (2%)
Constipation	2 (4%)
Dizziness	1 (2%)
Dyspepsia	2 (4%)
Nasal congestion	1 (2%)
Nausea	1 (2%)

## Discussion

In the present study, we evaluated the efficacy and safety of mirabegron monotherapy in very older (>80 years) women with OAB who were discontinued anticholinergic drugs by the other departments. We found that mirabegron monotherapy in elderly patients was an effective and safe treatment choice in a 12-week period for the management of OAB.

In cases where behavioral treatments are unsuccessful or insufficient in the treatment of OAB, pharmacotherapy is initiated, and mirabegron or antimuscarinic agents are recommended [13], although mirabegron may be used as an alternative medical treatment agent in cases where antimuscarinic agents are insufficient and especially in the patient group with high side effects. In the last 30-35 years, no drug group different from antimuscarinics has been developed, and mirabegron was the first agent developed and licensed in this area [14]. Chapple et al., in a study of 314 patients, investigated the reduction in voiding frequency over a four-week period using a placebo, mirabegron 50 mg, mirabegron 100 mg, and tolterodine. At the end of four weeks, it was reported that both doses of mirabegron provided a significant improvement compared to the placebo [15]. In a systematic review by Maman et al. in 2014, they compared mirabegron with all antimuscarinic agents. In their review study, it was determined that 50 mg mirabegron was similar to antimuscarinic agents in terms of 24-hour voiding and urinary incontinence attacks [16]. In the SCORPIO study, the efficacy of mirabegron therapy was compared with placebo. It was then compared with tolterodine and placebo in the same study. Mirabegron 50 mg significantly improved the number of urinary incontinence episodes and voiding frequency over a 24-hour period compared to placebo. With the placebo, the number of 24-hour urinary incontinence episodes decreased by 1.17, mirabegron by 1.57, and tolterodine by 1.27 [17]. A total of 928 patients were evaluated in the DRAGON study, which examined the dose-dependent efficacy of mirabegron in the treatment of OAB. When the responses obtained with mirabegron treatment at different doses (25, 50, 100, 150, and 200 mg) were examined, a significant improvement in voiding frequency was obtained with doses of 50, 150, and 200 mg mirabegron [18]. In our study, similar to the current literature, we found that mirabegron monotherapy (in a 12-week period) in very older patients decreased symptom scores and episodes of nocturia, frequency, and urgency. Significant differences were recorded in all of these values before and after mirabegron monotherapy (p < 0.05).

Mirabegron 50 mg extended-release (ER) form has a similar side effect profile to placebo. The most common side effects of antimuscarinic agents are dry mouth, constipation, and cognitive dysfunctions. These are less common in mirabegron therapy, and they were found at similar rates to placebo. Nitti et al. compared the side effects of placebo, mirabegron, and tolterodine ER form treatments. They reported rates of dry mouth at 1.6%, 0.9%, and 9.5% in patients using placebo, mirabegron 50 mg, and tolterodine, respectively. For constipation, these rates were found at 1.4%, 1.6%, and 2%, respectively. Changes in hypertension and heart rate were reported to be similar in all three agents [19]. In a 12-week study, the cardiovascular side effects of mirabegron in patients aged 65 and over 75 years were examined, and no significant increase in heart rate was observed. Mirabegron-related cardiovascular side effects were found to be similar to tolterodine [20]. Severe cardiovascular side effects of mirabegron use have not been reported, and this agent is not recommended only in patients with severe uncontrolled hypertension (systolic blood pressure > 180 mmHg and/or diastolic blood pressure > 110 mmHg). Antimuscarinic agents may cause central nervous system side effects such as sleepiness, fatigue, confusion, delirium, and cognitive impairment. One of the most important advantages of mirabegron, a beta-3 agonist, is that it does not pose a cognitive risk, and it does not create an anticholinergic load in sensitive and elderly individuals. Wagg et al. reported that mirabegron is a potential alternative to antimuscarinics in oral pharmacotherapy for overactive bladder in elderly patients [20]. When evaluated in terms of its side effect profile, mirabegron does not show the side effects observed with anticholinergic agents. With this aspect, it is an alternative agent for the patient group with anticholinergic load, the elderly patient group, and patients who cannot use drugs due to side effects due to anticholinergic agents. Staskin et al., in an eight-week study, examined the preferences of patients with OAB for mirabegron and tolterodine and the side effect profiles of these drugs. The drug compliance and tolerability index were found to be significantly higher in the mirabegron group, but no difference was observed in terms of drug preference. Improvement in symptoms was also similar in both groups [21]. In the present study, no significant cardiac side effects or changes of cardiac parameters (heart rate, systolic blood pressure, and diastolic blood pressure) occurred, and it was observed that mirabegron monotherapy was well tolerated by the very older patients. Also, in our study, we did not observe renal failure, liver transaminase elevations, QT prolongation, irregular heart rhythms, urinary retention, and drug interaction in any patients.

Our study contained some limitations. First, it was a retrospective study. Second, the sample sizes were small. Also, in our study, we evaluated short-term outcomes (a 12-week period). For a better understanding, studies that include extended-term outcomes are required. Finally, the present study did not include Mini-Mental State Examination outcomes.

## Conclusions

In conclusion, mirabegron is an effective and safe therapy in very older (>80 years) women with OAB. We recommend mirabegron monotherapy as an alternative medical treatment choice for the management of OAB. Prospective randomized trials with larger samples are required to achieve a better understanding.
